# Conservative Treatments Frequently Used for Chronic Pain Patients in Clinical Practice: A Literature Review

**DOI:** 10.7759/cureus.9934

**Published:** 2020-08-22

**Authors:** Min Cheol Chang

**Affiliations:** 1 Physical Medicine and Rehabilitation, College of Medicine, Yeungnam University, Daegu, KOR

**Keywords:** chornic pain, convervative treatment, medication, procedure, exercise

## Abstract

Chronic pain is a common patient complaint in clinical practice. It results in the deterioration of patients’ quality of life and loss of productivity. Also, it often brings about psychiatric disorders such as depression and anxiety. Therefore, clinicians should manage chronic pain actively. Various conservative treatments, including pharmacological therapy, procedures, and exercise, are being used to control chronic pain. In this review article, I provide an overview of the commonly used treatments, including medication [anticonvulsants, nonsteroidal anti-inflammatory drugs (NSAIDs), opioids, antidepressants], procedures [injection of steroids and local anesthetics, pulsed radiofrequency (PRF), repetitive transcranial magnetic stimulation (rTMS), prolotherapy], and exercise. A brief overview of these treatments would allow clinicians to have an overall picture of the available tools for managing chronic pain in clinical practice at a glance.

## Introduction and background

The most current definition of pain by the International Association for the Study of Pain (IASP) is “an unpleasant sensory and emotional experience associated with, or resembling that associated with, actual or potential tissue damage” [1]. Chronic pain is defined as pain suffered for longer than three months or after complete healing [2]. It may arise as a consequence of tissue damage, inflammation, and may have no identified cause. It has been reported that around 20% of the adult population suffers from chronic pain and in 8% of the individuals, the pain is so severe that it interferes with life or work activities [2]. Such chronic pain often brings about psychiatric disorders such as depression and anxiety. Chronic pain is not a simple extension of acute pain and can occur spontaneously without stimulus, while its degree is not proportional to that of the original damage. Such chronic pain can cause neural plasticity or sensitization of the nervous system in the peripheral nerve, spinal cord, and brain. In other words, it induces alterations to the nervous system [3]. Thus, chronic pain should be considered not as a mere symptom of disease, but as a disease in itself of the nervous system. To date, various methods have been applied to control chronic pain including exercise, physical therapy, medication, procedures, surgery, and psychotherapy [4-6]. In many cases, the symptoms of chronic pain are not fully resolved even with treatment, and a single mode of treatment often fails to control pain, necessitating the need for multiple treatment methods. This article aims to outline the conservative treatment methods often used in clinical practice to control chronic pain.

## Review

1. Medication

1.1. Anticonvulsants

Anticonvulsants are drugs used for the inhibition of seizures, which are also effective for neuropathic pain control [7]. Anticonvulsants control pain by inhibiting abnormal ectopic excitation in the injured nerve at a dose that does not inhibit the conduction of normal nerves. Since gabapentin and pregabalin are effective for pain control and cause only minor adverse reactions, they are often used in clinical practice. While both drugs have γ-aminobutyric acid (GABA)-like structures, they have no GABA-related activities and have no efficacy on the reuptake or metabolism of GABA [7]. They bind to the α2-δ subunit of the voltage-dependent calcium channels at synapses, which reduces the release of pain-related neurotransmitters [7]. Since these drugs are eliminated through the kidney, patients with a reduced renal function are likely to have adverse reactions such as severe dizziness or sedation, and the dose would therefore need to be reduced.

1.2. Nonsteroidal Anti-Inflammatory Drugs

Nonsteroidal anti-inflammatory drugs (NSAIDs) suppress inflammatory actions by the inhibition of cyclooxygenase (COX)-1/2, resulting in an analgesic effect [8]. While they are effective for the control of chronic pain related to higher inflammation, such as osteoarthritis and rheumatoid arthritis, long-term use is not advisable due to adverse reactions such as gastrointestinal bleeding and nephrotoxicity [8].

1.3. Opioids

Opioids include codeine, hydrocodone, and tramadol (not a true classic opioid with a week opioid agonist with serotonin-norepinephrine reuptake inhibitor properties). They induce an analgesic effect and adverse reactions through interactions with various opiate receptors [9]. Opioids are effective for persistent and dull pain rather than intermittent and sharp pain. Adverse reactions include nausea, vomiting, sedation, dizziness, and constipation. However, tramadol is not a true classic opioid.

1.4. Antidepressants

In addition to the treatment of mood disorders, antidepressants also have efficacy for pain control [10]. They inhibit the reuptake of neurotransmitters, such as serotonin or norepinephrine, and block potassium channels in the descending inhibitory pain pathway of the brain and spinal cord, which prevents the ectopic discharge of the injured nerve, leading to an analgesic action [10]. Antidepressants that are frequently used for the treatment of chronic pain in clinical practice include tricyclic antidepressants, selective serotonin reuptake inhibitors, and serotonin-norepinephrine reuptake inhibitors. These drugs are known to be effective mainly for neuropathy as well as tension headache, migraine, and fibromyalgia syndrome [10]. Adverse reactions of antidepressants include cardioplegia, arrhythmia, orthostatic hypotension, xerostomia, constipation, ischuria, vomiting, nausea, dizziness, and sedation, and are therefore prescribed at low doses initially, followed by a gradual increase.

2. Procedures

2.1. Injection of Steroids and Local Anesthetics

Intervertebral disc herniation or stenosis of the spine can cause inflammation or compression of the vertebral nerve, which often leads to chronic pain [11,12]. In addition, compression of peripheral nerves, as in carpal tunnel syndrome, tardy ulnar neuropathy, meralgia paresthetica, and tarsal tunnel syndrome, can be accompanied by neurogenic pain, often leading to chronic pain. A nerve blockade with a steroid and local anesthetics can effectively control such pain [11,12]. In addition, an injection of a steroid and local anesthetic into the joint can mitigate joint-origin pain [13]. This treatment has been known to block the transmission in nociceptive C-fiber and reduce ectopic discharge [11,12]. Moreover, steroids can control pain through the excitatory blockage of the peripheral nerve via the functional inhibition of inflammatory mediators [11,12]. Nerve blocks with steroids also inhibit the synthesis of various mediators of inflammation and reduce the inflammation of nerves induced by mechanical compression [11,12]. However, when nerves are severely compressed, injured, or damaged, the effectiveness of a nerve block with steroids and local anesthetics is reduced [11,12]. Recently, fluoroscopy- or ultrasound-guided injections have been utilized to accurately inject steroids into the site of pain [11,12]. Since repeated injections of steroids affect the secretion of adrenocortical hormones, besides causing adverse reactions such as elevation of blood sugar levels, elevated blood pressure, and osteoporosis, the number of administration must be controlled [13].

2.2. Pulsed Radiofrequency

Unlike conventional radiofrequency (RF) that controls pain through the ablation of nerves at a high temperature, pulsed radiofrequency (PRF) uses a needle tip under 42 °C by adding intervals between the electric stimuli to prevent the ablation of tissues [13]. PRF is applied not only for neurogenic pain, but also for various chronic pain disorders including muscle pain, joint pain, and discogenic pain [4,13]. While the mechanism of PRF in the control of chronic pain is yet to be understood, PRF is thought to alter pain transmission by inhibiting pain impulse propagation [4,13]. When PRF was applied to the dorsal root ganglia in a rat model of a herniated lumbar disc, microglial activity, involved in the generation and amplification of chronic pain, was reduced in the dorsal horn [4,13]. Because microglia release several cytokines and chemokines that are associated with progression to chronic pain, the down-regulation of their activities may control pain [14]. Additionally, PRF stimulation may cause microscopic damage to unmyelinated C-fibers that transfer pain sensation [15]. In 2016, Lee et al. compared the effects of PRF and a nerve root block with steroids on radicular pain due to a herniated disc [16]. They recruited 38 patients and randomly allocated them to PRF and steroid groups. After both procedures, radicular pain was significantly reduced, and it lasted for at least 12 weeks. During the 12-week period after each procedure, their pain-reducing effects were also similar. In 2020, Lee et al. performed PRF on cervical nerve roots in 49 patients who did not show a positive response to a nerve root block with a steroid, and they reported a significant pain reduction [17]. Therefore, PRF may be effective in patients who need frequent injections with steroids and in those who do not experience pain reduction after injection with steroids.

2.3. Repetitive Transcranial Magnetic Stimulation

Repetitive transcranial magnetic stimulation (rTMS) induces electric currents in the brain using high-frequency stimuli that have a short duration through a strong magnetic field [6]. At least 1,000 impulses below 1 ms are applied. It has been reported that rTMS is effective not only for neurogenic pain, but also for pain attributable to fibromyalgia syndrome, lumbar pain, and shoulder pain [6]. The primary motor cortex is the main target of stimulation. Previous functional magnetic resonance imaging studies have demonstrated that rTMS on the primary motor cortex induces changes in the activity of cortical and subcortical structures related to pain processing and modulation [6]. Furthermore, the application of rTMS on the primary motor cortex may influence the endogenous opioid system. However, most effective rTMS stimulation modes or sites, according to the types of chronic pain, have not been thoroughly elucidated. Further studies should clarify these.

2.4. Prolotherapy

Incomplete healing or partial damage to ligaments can result in secondary laxity and lability of the joint regulated by the ligament, ultimately resulting in chronic pain [18]. Prolotherapy refers to a treatment for such chronic pain in which a small amount of stimulant is injected into the ligament or tendon enthesis, inducing regeneration of new cells via inflammatory responses [18]. Stimulants are injected into ligaments attached to the bone or tendon enthesis, leading to inflammation, which subsequently induces the healing process. In other words, inflammatory mediators stimulate the secretion of growth factors that are directly helpful for healing, recruiting various cells, of which fibroblasts are the most important for the regeneration of the musculoskeletal system [18]. Regarding the treatment mechanism, these cells not only heal by inflammation but also actively produce collagen, inducing the regeneration of injured ligaments without healing, as well as tendon enthesis, which ultimately strengthens ligaments and tendon enthesis, leading to stability for pain control [18].

Usually, dextrose is used alone or in combination with other substances such as lidocaine or bupivacaine. Since it has been reported that at least 10% of dextrose is needed to induce inflammation, dextrose should be prepared at a concentration higher than 10%. However, concentrations of dextrose above 25% can cause problems such as the induction of severe pain or damage to proximal normal tissues; therefore, higher concentrations should be avoided. Thus, 10-25% of dextrose is generally used in prolotherapy [18].

3. Exercise

Chronic pain is likely to be accompanied by muscle atrophy, weakness, or joint contracture, which can be prevented by exercise. Chronic pain can also cause fatigue, inertia, and depression. Exercise increases the levels of adrenocortical hormones, cortisol, and catecholamine, which in turn produce beta-endorphins, resulting in reducing the sensation of pain [19]. Furthermore, exercise itself can be the most important treatment for some diseases. For example, muscle strengthening is the most fundamental treatment method for patellofemoral pain syndrome, attributable to the weakness of the vastus medialis [20]. As for lateral and medial epicondylitis, it is important to follow exercise regimens that strengthen the wrist extensor and the wrist flexor tendon, respectively. Also, in patients with fibromyalgia, exercise was reported to simulate brain regions involving in descending pain inhibition [21]. Many previous studies have reported that exercise can reduce the severity of pain, enhance physical function, and improve psychological function and quality of life [22]. However, these positive effects of exercise were not found in all studies [22]. This inconsistency could be due to the application of different types of exercise in each study and the quality of the studies. Therefore, further studies are needed on this topic.

## Conclusions

Various mechanisms are involved in the onset of chronic pain, even when the cause is identical, and various types of pain can be induced. Thus, effective treatment methods are dependent on each individual's needs, and in most cases, chronic pain can be more effectively controlled by a combination of various treatment methods. In addition to the aforementioned treatment methods to reduce chronic pain, various other methods have also been attempted, and effective outcomes have been consequently reported. Clinicians should be aware of currently employed treatment methods for each type of chronic pain to most effectively treat patients. Efforts should also be made to update the knowledge of clinicians about the innovative treatment methods that are being developed, followed by their application in patients.
